# Prevalence of Accommodative Microfluctuations in Eyes after Cataract Surgery

**DOI:** 10.3390/jcm12155135

**Published:** 2023-08-05

**Authors:** Tomoko Kaida, Takashi Ono, Tadatoshi Tokunaga, Keita Takada, Shota Tokuda, Naoto Kuwabara, Takushi Kawamorita, Kazutaka Kamiya, Nobuyuki Shoji, Kazunori Miyata

**Affiliations:** 1 Department of Ophthalmology, Miyata Eye Hospital, Miyazaki 885-0051, Japantokunaga@miyata-med.ne.jp (T.T.); tokuda@miyata-med.ne.jp (S.T.);; 2 Department of Ophthalmology, Graduate School of Medicine, University of Tokyo, Tokyo 113-8654, Japan; 3 Department of Orthoptics and Visual Science, Kitasato University School of Allied Health Sciences, Sagamihara 252-0373, Japan; 4 Department of Ophthalmology, School of Medicine, Kitasato University, Sagamihara 252-0374, Japan

**Keywords:** accommodative microfluctuations, accommodative spasms, high-frequency components, intraocular lens, pseudophakia

## Abstract

Background: We aimed to evaluate the existence of accommodative microfluctuations in eyes after cataract surgery. Methods: This retrospective observational cohort study included 1160 eyes of 713 patients (mean age: 72.5 ± 8.3 years) who underwent phacoemulsification, intraocular lens insertion, and an evaluation of accommodative microfluctuations with an autorefractometer. Patients with posterior segment disorders resulting in visual acuity impairment and those with unavailable medical information were excluded. High-frequency components (HFCs), between 1.0–2.3 Hz, based on fast Fourier transform analysis of the accommodative microfluctuation data were examined at postoperative 2–3 (2 M) and 6 months (6 M). The relationships between the HFCs and patient age, manifest refraction, and axial length were analyzed. Results: Increased HFC values (>65) were observed at a constant rate after cataract surgery, with prevalence rates of 33.4% at 2 M and 34.7% at 6 M. Postoperatively, at 2 M, increased HFC values were significantly more common for eyes with axial length ≥26 mm than for those with axial length <26 mm (*p* = 0.0056). However, they were not significantly correlated to age or postoperative manifest refraction. Conclusions: At 2 M postoperatively, increased HFC values presented more frequently in eyes with a greater axial length; hence, the precise detection and understanding of postoperative accommodative spasms in high myopia patients is important.

## 1. Introduction

Asthenopia, also known as ocular fatigue, is a common complaint of patients with computer vision syndrome [1,2,3]. The main causes of asthenopia are inappropriate accommodation and accommodative spasm [1,2]. For phakic eyes, accommodative spasm is often diagnosed as an imbalance of the ciliary muscle’s accommodative response to a real accommodative stimulus. Although asthenopia is a subjective symptom that is difficult to evaluate numerically, accommodative spasms can be objectively assessed using a medical device as accommodative microfluctuations [4,5,6]. Accommodative microfluctuations are rapid, small fluctuations in a refractive error that occur during sustained accommodation. Accommodative microfluctuations comprise two band patterns—namely, low-frequency components (LFCs), below 0.6 Hz, and high-frequency components (HFCs), between 1.0 and 2.3 Hz—based on the frequency analysis using the fast Fourier transform method. LFCs originate from a neurological source, while HFCs originate in the lens and its surrounding tissue [4,7]. When the ciliary muscles are tired, slight increases in the accommodative load can cause a high frequency. Thus, HFCs reportedly reflect accommodative spasms and are used as an index for asthenopia; however, they are affected by arterial pressure patterns [5,8]. Conventionally, the accommodative function is limited in eyes with an intraocular lens (IOL), suggesting that the occurrence of accommodative spasms is unlikely after cataract surgery. While the presence of an IOL may limit the amplitude of accommodation, some residual accommodation is possible in many cases. We have also encountered clinical cases of accommodative spasms after cataract surgery, even in the absence of any identifiable structural cause. Although an IOL position shift in pseudophakic eyes when accommodative stress is applied has been reported [9], no studies have evaluated accommodative microfluctuations in such eyes.

## 2. Materials and Methods

This observational study was approved by the Institutional Review Board of Miyata Eye Hospital, Miyazaki, Japan (identifier: CS-325), and performed in accordance with the tenets of the Declaration of Helsinki. The requirement of obtaining written informed consent was waived by the Institutional Review Board of Miyata Eye Hospital because the participants were provided the opportunity to opt out of the study.

### 2.1. Patients

Patients who underwent cataract surgery with the insertion of a monofocal IOL between January 2018 and October 2020 and whose accommodative function was evaluated 2–3 months (2 M) and 6 months (6 M) postoperatively were included in the study. Medical records were retrospectively reviewed with respect to the patient demographics, refractive and accommodative data, and axial length. Patients who had posterior segment disorders manifesting as visual acuity impairment and those with unavailable medical information at both examination points were excluded.

The axial length was measured using an OA-2000 instrument (TOMEY, Nagoya, Japan). The best corrected distance visual acuity was measured as the decimal visual acuity and converted to the logarithm of the minimum angle of resolution for calculations. Ocular refraction, expressed in the spherical and cylindrical lens power, was calculated as the spherical equivalent.

### 2.2. Accommodative Microfluctuation Analysis

Accommodative microfluctuations were evaluated in the same manner as previously reported [10]. The accommodative microfluctuation analysis was performed in a dimly lit examination room. The evaluation was performed by the same doctor (T.K.). In brief, the patients underwent examination using the autorefractometer Speedy-i (RIGHT MFG. Co., Ltd., Tokyo, Japan), and the accommodation response waveform was measured. Based on the refraction data at a far distance, changes in the objective refraction under various stimuli—which were set from +0.5 diopters (D) to −3.00 D in 0.5 D steps—were recorded. HFCs of the 1.0–2.3-Hz band were integrated at 0 D (resting position), −1 D, −2 D, and −3 D. Eyes with HFC values of >65 one or more times in response to 0, −1 D, −2 D, or −3 D stimulation were referred to as having increased HFC values, based on a previous report [2]. The pupil diameter was captured at the same time using Speedy-i.

### 2.3. Cataract Surgical Procedure

The general preoperative physical status of the patients was determined as stable by a physician, and their blood pressure was properly managed. Cataract surgery was performed under topical anesthesia with 4% xylocaine instillation. A corneal or sclerocorneal incision was made, and a subconjunctival injection of 4% xylocaine (0.5 mL) was administered in cases of sclerocorneal incision. After phacoemulsification and aspiration with the Centurion system (Alcon Laboratories, Fort Worth, TX, USA), the IOL was fixed within the capsule and the wound was closed. The patients were then administered a subconjunctival injection of dexamethasone (0.3 mL), levofloxacin (1.5%) eyedrops, and ofloxacin ointment. The postoperative medications were 1.5% levofloxacin eye drops 4 times/day for 1 week, 0.1% betamethasone sodium phosphate eye drops 4 times/day for 2 weeks, and 0.1% bromfenac sodium eye drops 2 times/day for 12 weeks. Cataract surgery was performed by multiple professional doctors at Miyata Eye Hospital. Several types of IOLs were used, depending on the surgeon.

### 2.4. Statistical Analyses

Statistical analyses were performed using Bell Curve for Excel (Social Survey Research Information, Tokyo, Japan). After the test for normality, the Kruskal–Wallis test with Steel–Dwass multiple comparison was conducted to compare the HFCs with each stimulus. The Wilcoxon signed-rank test was used to compare the HFCs between the 2 M and 6 M time points. The Cochran–Armitage test was employed to examine the relationship between increased HFC values and patient age. A linear regression model was applied to compare the HFC values with the patients’ age and manifest refraction. A chi-square test was performed to compare the ratio of increased HFC values depending on the axial length. Multiple linear regression analyses were performed to determine the factors affecting the HFC, with the HFC values as objective variables and axial length, age, anterior chamber depth, preoperative visual acuity, operation time, and preoperative manifest refraction as explanatory variables. Data are presented as mean ± standard deviation. Statistical significance was set at *p* < 0.05.

## 3. Results

### 3.1. Patient Characteristics

A total of 1160 eyes (575 right eyes, 585 left eyes) of 713 patients with a mean age of 72.5 ± 8.3 (31–91) years were included in the study. The patient characteristics are shown in Table 1. Cataract surgery significantly improved the best corrected distance visual acuity (*p* < 0.001). The number of patients with increased HFC values in the ≤49, 50–59, 60–69, 70–79, and ≥80 years age groups was 14 (1.2%), 50 (4.3%), 272 (23.4%), 600 (51.7%), and 224 (19.3%), respectively.

**Figure 1:**
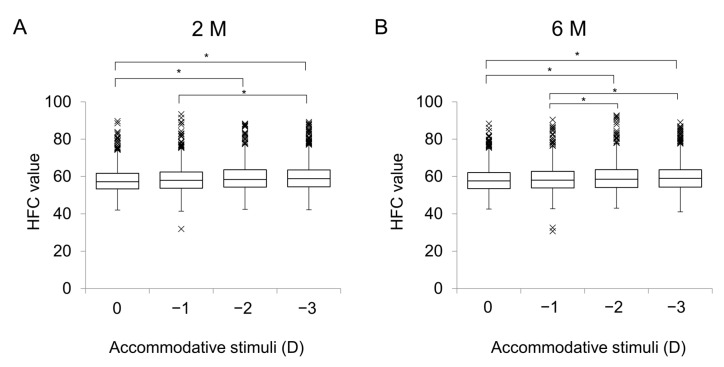
High-frequency component values for various accommodative stimuli in patients after cataract surgery. (**A**) HFC values for accommodative stimuli at 2 M. There were significant differences between 0 D and −2 D (*p* < 0.001), 0 D and −3D (*p* < 0.001), and −1 D and −3 D (*p* < 0.001). (**B**) HFC values for accommodative stimuli at 6M. There were significant differences between 0 D and −2 D (*p* < 0.001), 0 D and −3 D (*p* < 0.001), −1 D and −2 D (*p* = 0.032), and −1D and −3 D (*p* = 0.0031). Abbreviations: D, diopters; HFC, high-frequency component; M, months. ×, outliers; *, *p* < 0.05.

For the 0 D, −1 D, −2 D, and −3 D stimuli, the mean HFC values were 58.0 ± 6.6, 58.4 ± 6.9, 59.1 ± 6.9, and 59.5 ± 7.4, respectively, at 2 M, and 58.2 ± 6.7, 58.6 ± 7.0, 59.4 ± 7.3, and 59.6 ± 7.3, respectively, at 6 M (Figure 1A,B). At 2M, there were significant differences in the HFC values between 0 D and −2 D (*p* < 0.001), 0 D and −3 D (*p* < 0.001), and −1 D and −3 D (*p* = 0.0057). At 6 M, there were significant differences in the HFC values between 0 D and −2 D (*p* < 0.001), 0 D and −3 D (*p* < 0.001), -and −1 D and −3 D (*p* = 0.014). There were no differences in the HFC values for each accommodative stimulus between 2 M and 6 M (all *p* > 0.05).

### 3.2. HFCs and Age/Manifest Refraction

**Figure 2:**
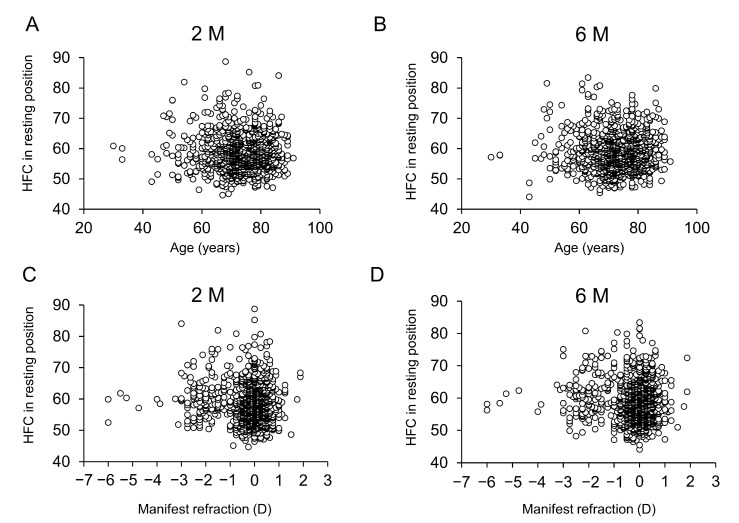
Relationship of high-frequency component values with patient age and manifest refraction after cataract surgery. Relationship between HFC values and patient age at 2 M (**A**) and 6 M (**B**). HFC values based on the patient age are plotted (○) (**A**,**B**). HFC values based on the manifest refraction are plotted (○) (**C**,**D**). There was no significant difference in HFC values according to age at either time point (A: r = −0.022, *p* = 0.462, B: r = 0.003, *p* = 0.912). Relationship between HFC values and manifest refraction at 2M (**C**) and 6 M (**D**). There was no significant difference in HFC values according to manifest refraction at either time point (C: r = −0.002, *p* = 0.940, D: r = −0.017, *p* = 0.569).

There was no correlation between the HFC values at the resting state of accommodation examined at 2 M and 6 M and the patients’ age (Figure 2A, B) or postoperative manifest refraction (Figure 2C,D).

### 3.3. Pupil Diameter

At 2M, the mean pupil diameters were 5.00 ± 1.51, 5.00 ± 1.51, 4.97 ± 1.52, and 4.95 ± 1.52 mm for the 0 D, −1 D, −2 D, and −3 D stimuli, respectively, with no significant difference among the accommodative stimuli. At 6 M, the pupil diameters were 5.52 ± 1.50, 5.49 ± 1.52, 5.46 ± 1.52, and 5.41 ± 1.52 mm for the 0 D, −1 D, −2 D, and −3 D stimuli, respectively, there was also no significant difference among the accommodative stimuli.

### 3.4. Prevalence of Increased HFC Values

The prevalence of increased HFC values (>65) after cataract surgery at least once for various accommodative stimulations was 33.4% (388 eyes) at 2 M and 34.7% (403 eyes) at 6 M, with no significant difference between 2 M and 6 M.

**Figure 3:**
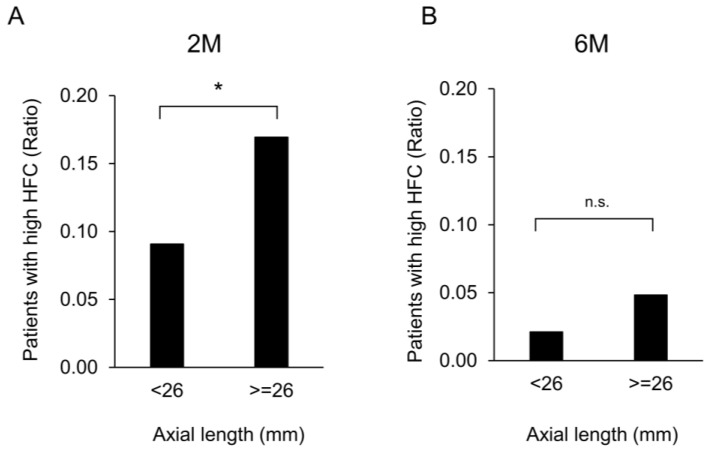
Ratio of patients with increased high-frequency component values depending on the axial length of eyes after cataract surgery. (**A**) Comparison of the ratio of patients with increased HFC values at axial length <26 and ≥26 mm at 2 M. Increased HFC values were significantly more frequent in eyes with an axial length ≥ 26 mm than in those with an axial length <26 mm at 2 M (*p* = 0.0056). (**B**) Comparison of the ratio of patients with increased HFC values at axial length < 26 and ≥ 26 mm at 6 M. There was no significant difference between eyes with axial lengths ≥26 mm and <26 mm at 6 M. Abbreviations: n.s.: not significant. *, *p* < 0.05.

Eyes with an axial length of ≥26 mm and those with an axial length of <26 mm were observed in 124 and 1036 patients, respectively (65.2 ± 8.0 and 73.5 ± 7.6 years, respectively, *p* < 0.001). Increased HFC values were significantly more common in eyes with a long axial length (≥26 mm) than in eyes with an axial length of <26 mm at 2 M (*p* = 0.0056) (Figure 3A). However, there was no significant difference in this relationship at 6 M (Figure 3B). Furthermore, multiple linear regression confirmed that only the axial length was significantly related to the HFC value at 2 M (*p* = 0.003); however, there was no significant relation at 6 M.

The age distribution of the patients with increased HFC values is presented in Table 2. The Cochran–Armitage test showed no significant trend regarding the ratio of patients with increased HFC values and their age examined at both 2 M and 6 M. There was no significant difference in the manifest refraction between patients with and those without increased HFC values at both 2 M and 6 M.

## 4. Discussion

In this study, we demonstrated that increased HFC values were constantly observed 2 M and 6 M after cataract surgery, and increased HFC values presented more frequently in eyes with a greater axial length. The presence of an increased HFC is considered the reflection of postoperative asthenopia.

HFCs reflect accommodative spasm [2]. After cataract surgery, it is believed that no accommodative reaction occurs because of the loss of accommodative changes in the lens. However, we sometimes encounter patients who complain of asthenopia of unknown origin after cataract surgery. In a previous study, Lesiewska-Junk et al. [11] reported that the IOL moved forward 0.42 mm for near vision in young patients after cataract surgery, resulting in adjustment impairment. In the present study of more than 1000 eyes, we also observed the occurrence of increased HFC values in pseudophakic eyes, suggesting that slight IOL movement occurs even after cataract surgery; this finding is the core of our results. The accommodative stimulation of an eye with a monofocal IOL can cause an anterior shift of the lens capsule and changes in the ciliary process.

Changes in the IOL intraocular position depending on the accommodative stimulus have been the focus of recent research [9]. Ciliary body movement during accommodative effort can be observed even after cataract surgery to change the shape of the optical structure and adjust the focus. Win-Hall and Glasser [9] found that eyes with a monofocal IOL demonstrated a difference between the subjective and objective refractive values for near vision, suggesting the presence of accommodative effort, although the accommodative capacity was nearly zero. In phakic eyes, the degree of asthenopia is objectively evaluated by measuring the accommodative refractive changes and accommodative microfluctuations, which reflect the swaying and instability of the eyeball caused by ciliary muscle contraction in near vision. In this process, a complex combination of the effects of ocular aberrations, depth of field of the eye, and psychophysical blur perception are considered to be related [9]. In the present study, our results showed no correlation between the subjective refraction values and HFC values, suggesting no influences from these factors. LFCs reportedly reflect regulation involving neurogenic changes, while HFCs increase with accommodative stimulation [1]. Lupón et al. [12] found that noise from the arterial blood flow and orbital movements is related to HFCs, and Rendondo et al. [13] reported that HFCs are influenced by heart rate and body movement. In this clinical study, we evaluated HFCs as an index for accommodative changes in pseudophakic eyes, and the significance of HFCs in accommodation regulation should be further reinforced.

When the ciliary muscle is fatigued, HFCs increase because of the regulatory load in the phakic eye [2]; however, there have been no reports regarding accommodative microfluctuations in eyes with an IOL. Our results indicated that increased HFC values were present in a certain percentage of eyes with an IOL. Further clinical studies are required to analyze the relationship between increased HFC values and asthenopia in eyes with IOLs. In contrast, the average HFC values in the resting position examined in our study were almost the same as the HFC values for phakic eyes in an earlier study [2]. The HFC values were also the same at 2 M and 6 M as they were at the resting position; this result is in agreement with the findings of a previous study by Win-Hall and Glasser [9], indicating that the accommodative capacity of monofocal IOL eyes after cataract surgery is almost zero.

Compared with older patients, younger patients present with greater apparent accommodation when the lens shifts due to the contraction of the ciliary muscle caused by accommodative stimuli. Thus, it was expected that the HFC values would be higher for younger individuals and for those with a greater refractive error; however, no correlation was found between the HFC values and age or manifest refraction in our results. In contrast, increased HFC values were significantly more common in eyes with a long axial length, which included younger patients, and this was demonstrated through both the univariate and multivariate analyses. It has been previously reported that ocular fatigue is observed in patients after cataract surgery [14]. Further, based on our findings, in clinical settings, patients with high myopia complain of ocular fatigue after cataract surgery. Ocular fatigue symptoms could be overlooked in patients after cataract surgery; therefore, ophthalmologists should be mindful of this phenomenon. This result is reinforced by a previous study that demonstrated a larger accommodative lag in myopic eyes [15] and another study that showed that the HFC values gradually increased with sustained near work [16]. Additionally, our result may be attributable to patients having been forced to see objects with the untreated eye after unilateral surgery. The fact that the HFC values in eyes with a greater axial length were significantly higher at 2 M and at the same level at 6 M suggests that some kind of adaptive systems work only in eyes with a long axial length. In particular, cataract surgery with proper IOL reduces the degree of myopia in patients with preoperative high myopia, and the refractive change from the preoperative state is large in high myopia. Because those with a long axial length were significantly younger, this adaptation system—which is more predominant in young patients—might have contributed to this phenomenon.

Wagner et al. [17] reported a difference in the thickness of the ciliary muscle during accommodation between normal and myopic eyes, showing that it was thicker in myopic than in normal eyes. Therefore, it has been speculated that the greater the thickness of the ciliary muscle, the greater the width of its accommodative movement, and the greater the movement of the lens capsule where the IOL is inserted. Our data demonstrated that the pupil diameter was not different for each accommodation stimulus, although we did not measure the ciliary muscle thickness in the current study.

Dry eye is reportedly associated with accommodative microfluctuations [18]. Dry eye could result in ocular symptoms, such as ocular itching, foreign body sensation, redness, hyperemia, and visual disturbance. Further, dry eye exacerbates presbyopia, results in weakened accommodative function with aging [19], and is often observed to be exacerbated after cataract surgery [20]. In the current study, we did not evaluate the degree of dry eye in the included patients. However, future studies should evaluate postoperative dry eye using multivariate analysis.

This study had several limitations. First, because of its retrospective nature, we did not simultaneously assess the three principles of accommodation: refractive changes, convergent ocular position, and pupil constriction. Because these data could be confounding factors, in order to accurately assess the relationship between increased HFC values and regulatory spasm, a prospective study including more examination items, such as convergence and IOL data, is required. Additionally, there was selection bias owing to the retrospective setting, limited statistical analyses, and missing data. Second, because HFCs could be related to arterial pressure patterns, general circulatory dynamics should be evaluated. However, all of the patients that we included were properly assessed and treated by a physician preoperatively, and their general status was considered to be stable. Finally, there remained a logical discrepancy between accommodative fluctuations and refractive changes, possibly because of IOL movement. We only examined HFCs, and it will also be necessary to analyze LFCs of pseudophakic eyes in future studies. In this study, we evaluated patients after unilateral cataract surgery, as well as those after bilateral cataract surgery. Considering the impact of the binocular function on eye strain, these two groups should be separately evaluated; however, we could not obtain all the information regarding the non-operated eye of the unilateral cataract surgery patients. This issue should be analyzed in a future study. Further, the accommodation measurement device used in the present study measures the eye unilaterally, using the internal optotype. This method does not always accurately assess the accommodative response compared with dynamic retinoscopy, which can test eyes under binocular vision. However, dynamic retinoscopy requires skilled techniques, making it difficult to practically test a large number of participants.

## 5. Conclusions

In conclusion, increased HFC values were observed in 30%–40% of the eyes with monofocal IOL. Increased HFC values were observed more frequently in patients with eyes that had a long axial length, and adaptation occurred over time. It is important to consider postoperative accommodative spasms in patients with high myopia.

## Figures and Tables

**Table 1:** Patient characteristics.

N (Eyes)	1160
Age (years)	72.5 ± 8.3
Preoperative anterior chamber depth (mm)	3.18 ± 0.41
Axial length (mm)	23.99 ± 1.57
Preoperative best corrected distance visual acuity (logMAR)	0.21 ± 0.27
Postoperative best corrected distance visual acuity (logMAR)	−0.08 ± 0.12
Preoperative manifest refraction (D)	−1.29 ± 3.68
Postoperative manifest refraction (D)	−0.11 ± 5.2
Preoperative corneal endothelial cell density (cells/mm^2^)	2631 ± 331
Postoperative corneal endothelial cell density (cells/mm^2^)	2594 ± 714
Surgical time (s)	611.6 ± 319.0

Data are presented as mean ± standard deviation. Abbreviations: D, diopters; logMAR, logarithm of the minimum angle of resolution.

**Table 2:** Age distribution of patients with increased high-frequency component values.

Age Group (Years)	N	2 M (Eyes)	6 M (Eyes)
≤49	14	7 (50%)	6 (43%)
50–59	50	16 (32%)	20 (40%)
60–69	272	101 (37%)	90 (33%)
70–79	600	186 (31%)	204 (34%)
≥80	224	76 (34%)	85 (38%)
*p*-value	-	0.35	0.54

Abbreviations: M, months.

## Data Availability

The data generated during the current case series are available from the corresponding author on reasonable request.

## References

[1] Blehm C., Vishnu S., Khattak A., Mitra S., Yee R.W. (2005). Computer vision syndrome: A review. Surv. Ophthalmol..

[2] Kajita M., Ono M., Suzuki S., Kato K. (2001). Accommodative microfluctuation in asthenopia caused by accommodative spasm. Fukushima J. Med. Sci..

[3] Lee S.H., Kim M., Kim H., Park C.Y. (2021). Visual fatigue induced by watching virtual reality device and the effect of anisometropia. Ergonomics.

[4] Charman W.N., Heron G. (1988). Fluctuations in accommodation: A review. Ophthalmic Physiol. Opt..

[5] Winn B., Pugh J.R., Gilmartin B., Owens H. (1990). The frequency characteristics of accommodative microfluctuations for central and peripheral zones of the human crystalline lens. Vis. Res..

[6] Campbell F.W., Robson J.G., Westheimer G. (1959). Fluctuations of accommodation under steady viewing conditions. J. Physiol..

[7] van der Heijde G.L., Beers A.P., Dubbelman M. (1996). Microfluctuations of steady-state accommodation measured with ultrasonography. Ophthalmic Physiol. Opt..

[8] Collins M., Davis B., Wood J. (1995). Microfluctuations of steady-state accommodation and the cardiopulmonary system. Vis. Res..

[9] Win-Hall D.M., Glasser A. (2009). Objective accommodation measurements in pseudophakic subjects using an autorefractor and an aberrometer. J. Cataract. Refract. Surg..

[10] Kajita M., Muraoka T., Orsborn G. (2020). Changes in accommodative micro-fluctuations after wearing contact lenses of different optical designs. Contact Lens Anterior Eye.

[11] Lesiewska-Junk H., Kałuzny J. (2000). Intraocular lens movement and accommodation in eyes of young patients. J. Cataract. Refract. Surg..

[12] Lupón N., Gispets J., Cardona G., Tàpia A., Abril H. (2019). Role of microfluctuations in accommodation: A novel approach to reduce non-accommodative noise. Int. J. Ophthalmol..

[13] Redondo B., Vera J., Luque-Casado A., García-Ramos A., Jiménez R. (2019). Associations between accommodative dynamics, heart rate variability and behavioural performance during sustained attention: A test–retest study. Vis. Res..

[14] Hanyuda A., Ayaki M., Tsubota K., Negishi K. (2019). Discrepancies in persistent dry eye signs and symptoms in bilateral pseudophakic patients. J. Clin. Med..

[15] Chen A.H., Ahmad A., Kearney S., Strang N. (2019). The influence of age, refractive error, visual demand and lighting conditions on accommodative ability in Malay children and adults. Graefe’s Arch. Clin. Exp. Ophthalmol..

[16] Yu H., Zeng J., Li Z., Hu Y., Cui D., Zhao W., Zhao F., Yang X. (2022). Variability of accommodative microfluctuations in myopic and emmetropic juveniles during sustained near work. Int. J. Environ. Res. Public Health.

[17] Wagner S., Zrenner E., Strasser T. (2019). Emmetropes and myopes differ little in their accommodation dynamics but strongly in their ciliary muscle morphology. Vis. Res..

[18] Kaido M., Kawashima M., Shigeno Y., Yamada Y., Tsubota K. (2017). Relation of accommodative microfluctuation with dry eye symptoms in short tear break-up time dry eye. PLoS ONE.

[19] Ayaki M., Negishi K. (2022). Short tear breakup time could exacerbate the progression of presbyopia in women. BioMed Res. Int..

[20] Naderi K., Gormley J., O’Brart D. (2020). Cataract surgery and dry eye disease: A review. Eur. J. Ophthalmol..

